# PKC-ALDH2 Pathway Plays a Novel Role in Adipocyte Differentiation

**DOI:** 10.1371/journal.pone.0161993

**Published:** 2016-08-30

**Authors:** Yu-Hsiang Yu, Pei-Ru Liao, Chien-Jung Guo, Che-Hong Chen, Daria Mochly-Rosen, Lee-Ming Chuang

**Affiliations:** 1 Department of Biotechnology and Animal Science, National Ilan University, Ilan, Taiwan; 2 Department of Internal Medicine, National Taiwan University Hospital, Taipei, Taiwan; 3 Institute of Molecular Medicine, College of Medicine, National Taiwan University, Taipei, Taiwan; 4 Department of Chemical and Systems Biology, Stanford University School of Medicine, Stanford, California, 94305, United States of America; 5 Department of Internal Medicine, College of Medicine, National Taiwan University, Taipei, Taiwan; University of Cambridge, UNITED KINGDOM

## Abstract

The *ALDH2* gene encodes the mitochondrial aldehyde dehydrogenase 2 (ALDH2), a critical enzyme involved in ethanol clearance through acetaldehyde metabolism. ALDH2 also catalyzes the metabolism of other bioreactive aldehydes, including propionaldehyde, butyraldehyde, and 4-hydroxykenals (4-HNE). Increased levels of 4-HNE in adipose tissue positively correlate with obesity and insulin resistance. However, it remains unclear whether ALDH2 is involved in regulation of adipocyte differentiation. Here, we found that ALDH2 protein levels were lower in white adipose tissue of high-fat diet-fed mice and *ob/ob* mice relative to lean mice. Knockdown of ALDH2 expression in 3T3-L1 preadipocytes caused an increase in intracellular 4-HNE, thereby attenuated adipocyte differentiation. By contrast, an ALDH2 activator, Alda-1, significantly accelerated adipogenesis, which was accompanied by an increase in adipogenic gene expression. Consistently, adipogenesis was reduced when protein kinase C ε (PKCε), an ALDH2 phosphorylating activator, was silenced in 3T3-L1 preadipocytes, whereas treatment with a PKCε agonist in 3T3-L1 preadipocytes enhanced adipogenesis. Whole-genome microarray profiling of Alda-1-treated cells demonstrated several upregulated transcripts encoding proteins involved in metabolism and the majority of these transcripts are for proteins involved in PPAR signaling pathways. Furthermore, PKCε-ALDH2 interaction alleviates 4-HNE induced aberrant PPARγ regulation on adipogenesis. Taken together, these results demonstrate that ALDH2 activation enhances adipogenesis and signaling pathways involving PPARγ. Thus, activation of PKCε-ALDH2 regulatory axis may be a therapeutic target for treating obesity and type 2 diabetes.

## Introduction

Obesity and type 2 diabetes have become global epidemic with huge impact on human health. Emerging evidence shows oxidative stress is closely associated with metabolic syndromes [1]. 4-hydroxynonenal (4-HNE), a reactive aldehydes generated from lipid peroxidation, accumulates in numerous oxidative stress-related diseases, including obesity [2, 3]. Several proteins involved in lipid metabolism are carbonylated by 4-HNE, leading to modification of cell signaling [4, 5]. Increased intracellular 4-HNE-protein adducts formation in adipocytes induce lipolytic response, indicating that 4-HNE have a relevant biology role in the differentiated adipocytes [6]. In addition, 4-HNE accumulation can impair aldehyde detoxification, by inactivating key enzymes such as aldehyde dehydrogenase 2 (ALDH2) [7].

The *ALDH2* gene encodes the mitochondrial ALDH2, a critical enzyme not only for oxidation of acetaldehyde to acetic acid in ethanol metabolism, but also for 4-HNE metabolism [8]. ALDH2 activity increases *via* direct phosphorylation by protein kinase C ε (PKCε) [9]. ALDH2 activator, Alda-1, is able to increase the catalytic activity of ALDH2 [9]. Structural analysis also demonstrated that Alda-1 is a chemical chaperone that binds to the substrate tunnel of ALDH2 [10].

It has been reported that ALDH2 expression negatively correlates with obesity in mice [11] while 4-HNE accumulation is positively associated with obesity and is increased in terminal adipocyte differentiation [6]. However, whether PKCε-ALDH2 regulatory axis mediates adipogenesis through metabolism of 4-HNE has not yet been determined. Therefore, we assessed the effects of PKCε and ALDH2 on adipocyte differentiation.

## Materials and Methods

### Mice

Eight male C57BL/6 mice at 4-weeks of age were randomly allocated to two different treatment groups (n = 4 per group): standard mouse chow diet group (13% calorie from fat, TestDiet 5010) and HFD group (60% calorie from fat, TestDiet 58Y1). Four male C57BL/6 (4-week-old) mice and four male *ob/ob* (4-week-old) mice fed a standard mouse chow diet group (13% calorie from fat, TestDiet 5010). All animal protocols were approved by the Animal Care and Use Committee of the National Taiwan University. Mice were housed at 23°C and light/dark cycles of 12/12 h. Water and feed was provided ad libitium throughout two months of the entire experimental period. After two months, mice were sacrificed by cervical dislocation with anesthesia (tribromoethanol, 0.4 mg/g of body weight, i.p.; Sigma, St. Louis, MO).

### Cell culture

All chemical reagents were purchased from Sigma-Aldrich (St. Louis, MO) unless specified otherwise. Alda-1 was provided from ALDEA Pharmaceuticals. PKCε agonist, RACK [12] was synthesized and purified at the Stanford Protein and Nucleic Acid Facility. The 3T3-L1 preadipocytes were cultured in Dulbecco’s modified Eagle medium (DMEM) with 10% calf serum (CS) at 37°C in an atmosphere of 5% CO_2_ in air. After confluence, 3T3-L1 preadipocytes were then cultured in induction medium (DMEM containing 10% FBS, 1 μM dexamethasone, 0.5 mM 3-isobutyl-1-methylxanthine and 5 μg/mL insulin). After 2 days, the cells were maintained in DMEM containing 10% FBS and 5 μg/mL insulin. Two days later, cells were cultured in DMEM containing 10% FBS for 8 day with a medium change every 2 day. After 8 day of culture, cells on the plates were stained with Oil-Red O to measure the degree of adipocyte differentiation [13]. Cellular RNA was extracted to determine the mRNA concentrations for several genes whose expression increases during adipocyte differentiation: adipocyte fatty acid binding protein (FABP4), adiponectin (ADIPONECTIN), lipoprotein lipase (LPL) and acetyl CoA carboxylase (ACC).

### Lentiviral infection

Lentivirus carrying small hairpin RNA (shRNA) targeting mouse ALDH2, PKCε gene and the control vector (shLuc) were obtained from the National RNAi Core Facility of Academia Sinica in Taiwan. For gene silencing, confluent 3T3-L1 preadipocytes were infected with lentivirus carrying control shRNA, shRNA targeting ALDH2 or PKCε, respectively. One day after lentiviral infection, cells were selected by puromycin for 2 days. Two days later, single colonies were isolated for further propagation and medium was replaced with fresh medium containing puromycin every two days. Cells were selected with puromycin for at least 1 month. After 1 month selection, protein from cells is isolated and measured the ALDH2 and PKCε levels. For double knockdown, single lentiviral vector co-expressing shRNAs targeting ALDH2 and PKCε was used and selected by a selectable marker on puromycin. Detailed protocols for lentiviral infection of cells were performed following the procedures of the National RNAi Core Facility of Academia Sinica in Taiwan.

### Cell proliferation assay

Cell proliferation was measured by MTS assay (CellTiter 96 aqueous one solution cell proliferation assay) (Promega, Madison, WI) according to the manufacturer’s instructions. Briefly, 1x10^3^ cells were seeded in each well of 96-well microtiter plates. After 48 hour seeding, the percentage of viable cells was quantified by measuring the absorbance at 490 nm using a microtiter culture plate reader.

### Western blot

Protein extracts from tissue or cells were separated by sodium dodecyl sulfate-polyacrylamide gel electrophoresis and then transferred to a polyvinylidine fluoride membrane (Perkin Elmer, Norwalk, CT). For subcellular protein fractionation, we employed a kit purchased from Millipore (Billerica, MA) and followed the manufacturer’s instructions. The ALDH2 (no. ab108306), 4-hydroxynonenal (no. ab46545) and PPARγ (no. ab45036) primary antibody were purchased from Abcam (Cambridge, MA). PKCε (no. sc-214), α-tubulin (no. sc-32293) and cytochrome c (no. sc-13560) primary antibody were purchased from Santa Cruz Biotechnology (Dallas, TX). GAPDH (no. ab181602) and HSP70 (no. ab45133) antibody (Abcam, Cambridge, MA) were used for the loading control in the lysates of total protein. The secondary antibody coupled to horseradish peroxidase was used in the chemiluminescence procedure (Immobilon Western, Millipore, Billerica, MA). The Western blotting procedure was performed according to the manufacturer's instruction.

### Immunoprecipitation

3T3-L1 preadipocytes were harvested at the indicated times and lysed in NP40 cell lysis buffer (Invitrogen, Carlsbad, CA) for co-immunoprecipitation. Immunoprecipitations were carried out using antibodies coupled to Dynabeads Protein A (Dynabeads® protein A Immunoprecipitation Kit, Invitrogen, Carlsbad, CA) according to the manufacturer's instructions. Immunocomplexes were resolved on 12% SDS-PAGE and then immunoblotted. Antibodies used for immunoprecipitation were anti-ALDH2 (Abcam, Cambridge, MA), anti-4-hydroxynonenal (Abcam, Cambridge, MA) and anti-PKCε (Santa Cruz Biotechnology, Dallas, TX).

### Quantitative reverse transcription-PCR

Cellular RNA was extracted using TRIzol (Invitrogen, Carlsbad, CA). Reverse transcription was performed with a Transcriptor Reverse Transcriptase kit (Roche Applied Science, Indianapolis, IN). Quantitative reverse transcriptase-PCR was performed using ABI PRISM 7000 sequence detector system (Applied Biosystems, Foster City, CA) and KAPA SYBR® FAST qPCR Kit (Kapa Biosystems, Inc., Boston, MA). PCR was performed by 40 cycles of 95°C for 30 s, 58–60°C for 60 s, and 72°C for 30 s. 18S rRNA was determined as the internal control gene. The sequence of primers for quantitative reverse transcription-PCR was listed in Table 1. The mRNA expression of each gene was normalized to its 18S rRNA expression in the same sample. Threshold cycle (*C*_t_) values were obtained and relative gene expression was calculated using the formula (1/2)^Ct target genes−Ct 18S^.

**Table 1:** Primer sequences for quantitative reverse transcription-PCR.

Gene	Sequence	
ALDH2	Sense	5’- TTATCCAGCCCACCGTGTTC-3’
	Anti-sense	5’- GCTGCCAGCCCATACTTAGA-3’
FABP4	Sense	5’- GATGCCTTTGTGGGAACCTG-3’
	Anti-sense	5’- GCCATGCCTGCCACTTTC-3’
ADIPONECTIN	Sense	5’- TCCTGGAGAGAAGGGAGAGAAAG-3’
	Anti-sense	5’- CCCTTCAGCTCCTGTCATTCC-3’
LPL	Sense	5’-GGATGGACGGTAAGAGTGATTC-3’
	Anti-sense	5’-ATCCAAGGGTAGCAGACAGGT-3’
ACC	Sense	5’-AACATCCCCACGCTAAACAG-3’
	Anti-sense	5’-CTGACAAGGTGGCGTGAAG-3’
PPARγ	Sense	5’-CAAGAATACCAAAGTGCGATCAA-3’
	Anti-sense	5’-GAGCTGGGTCTTTTCAGAATAATAAG-5’
C/EBPα	Sense	5’-GAACAGCAACGAGTACCGGGTA-3’
	Anti-sense	5’-GCCATGGCCTTGACCAAGGAG-3’
C/EBPβ	Sense	5’-CAAGCTGAGCGACGAGTACA-3’
	Anti-sense	5’-CAGCTGCTCCACCTTCTTCT-3’
AQP7	Sense	5’-AGGCATTCGTGACTGGGATG-3’
	Anti-sense	5’-CACCCCAAGGACGGTAACAA-3’
ACOX2	Sense	5’-CTTGGCATGTTGGTGACACG-3’
	Anti-sense	5’-TCACTAGGCCGAAGACGAGA-3’
PCK1	Sense	5’-TGCCTCCTCAGCTGCATAAC-3’
	Anti-sense	5’-GGATATACTCCGGCTGGCAC-3’
NR1H3	Sense	5’-GGGATAGGGTTGGAGTCAGC-3’
	Anti-sense	5’-GGCCCTTTTTCCGCTTTTGT-3’
OLR1	Sense	5’-CATCCTCTGCCTGGTGTTGT-3’
	Anti-sense	5’-TCTGCCCTTCCAGGATACGA-3’
PREF-1	Sense	5’-GCGTGGACCTGGAGAAAG-3’
	Anti-sense	5’-GGAAGTCACCCCCGATGT-3’
18S	Sense	5’- ACGATGCCGACTGGCGATGC-3’
	Anti-sense	5’- TCCTGGTGGTGCCCTTCCGT-3’

Abbreviations used: aldehyde dehydrogenase 2, ALDH2; adipocyte fatty acid binding protein, FABP4; lipoprotein lipase, LPL; acetyl-CoA carboxylase, ACC; peroxisome proliferator-activated receptor γ, PPARγ; CCAAT-enhancer-binding protein α, C/EBPα; CCAAT-enhancer-binding protein β, C/EBPβ; aquaporin 7, AQP7; acyl-Coenzyme A oxidase 2, branched chain, ACOX2; phosphoenolpyruvate carboxykinase 1 (soluble), PCK1; nuclear receptor subfamily 1, group H, member 3, NR1H3; oxidised low density lipoprotein (lectin-like) receptor 1, OLR1.

### Microarray analysis

Total RNA from cultured cells was quantified using NanoDrop 1000 spectrophotometer (Thermo Fisher Scientific, Waltham, MA) and RNA integrity was evaluated using an Agilent 2100 bioanalyser (Agilent, Palo Alto, CA). RNA was amplified using an OneArray^**®**^ Amino Allyl aRNA Amplification Kit (Phalanx Biotech Group, Taiwan) and labeled with Cy5 dyes (Amersham Pharmacia, Piscataway, NJ). Labeled RNA was hybridized to the Mouse Whole Genome OneArray (Phalanx Biotech Group, Taiwan) and arrays were scanned by Axon 4000B scanner (Molecular Devices, Sunnyvale, CA). The Cy5 fluorescent intensities of each spot were analyzed by GenePix 4.1 software (Molecular Devices, Sunnyvale, CA). The signal intensity of each spot was loaded into Rosetta Resolver System^**®**^ (Rosetta Biosoftware) to process data analysis. The error model of Rosetta Resolver System^**®**^ could remove both systematic and random errors form the data. We filtered out spots that the flag is less than 0. Spots that passed the criteria were normalized by 50% media scaling normalization method. The technical repeat data was tested by Pearson correlation coefficient calculation to check the reproducibility (R value > 0.975). Normalized spot intensities were transformed to gene expression log_2_ ratios between the ethanol-treated and Alda-1-treated groups. The spots with log_2_ ratio ≥ 1 or log_2_ ratio ≤ -1 and *P*-value < 0.05 are tested for further analysis. Integrative extraction of frequently co-occurring annotations in a given gene list across different sources such as Gene Ontology terms and KEGG pathways was performed using GeneCodis software [14].

### Database submission of microarray data

The microarray data were deposited in the ArrayExpress database: www.ebi.ac.uk/arrayexpress. The ArrayExpress accession number for the platform is E-MTAB-4936.

### Transient transfection and reporter assay

For 293T cell transfection, confluent cells were cotransfected with plasmids (GAL4-PPARγ, UAS_G_×4-TK-LUC, Flag-ALDH2 and Flag-PKCε) and lipofectamine (Lipofectamine 3000, Invitrogen, Carlsbad, CA). After 6 h of transient transfection, media were changed to growth media with troglitazone, PKCε agonist and 4-HNE. Twenty-four hours after treatment, cells were harvested and luciferase activity was assayed by Dual-Glo luciferase assay system (Promega, Madison, WI).

### Ethics statement

All experiments were performed in accordance with the approved guidelines. The animal protocol was approved by the National Taiwan University Institutional Animal Care and Use Committee (IACUC Approval No. 20130384).

### Statistical analysis

Results are expressed as means ± SE. Comparison between two groups were performed using unpaired t-test. A significant difference indicated that the P-value was ≤0.05.

## Results

### ALDH2 is a positive regulator of adipocyte differentiation

**Fig 1:**
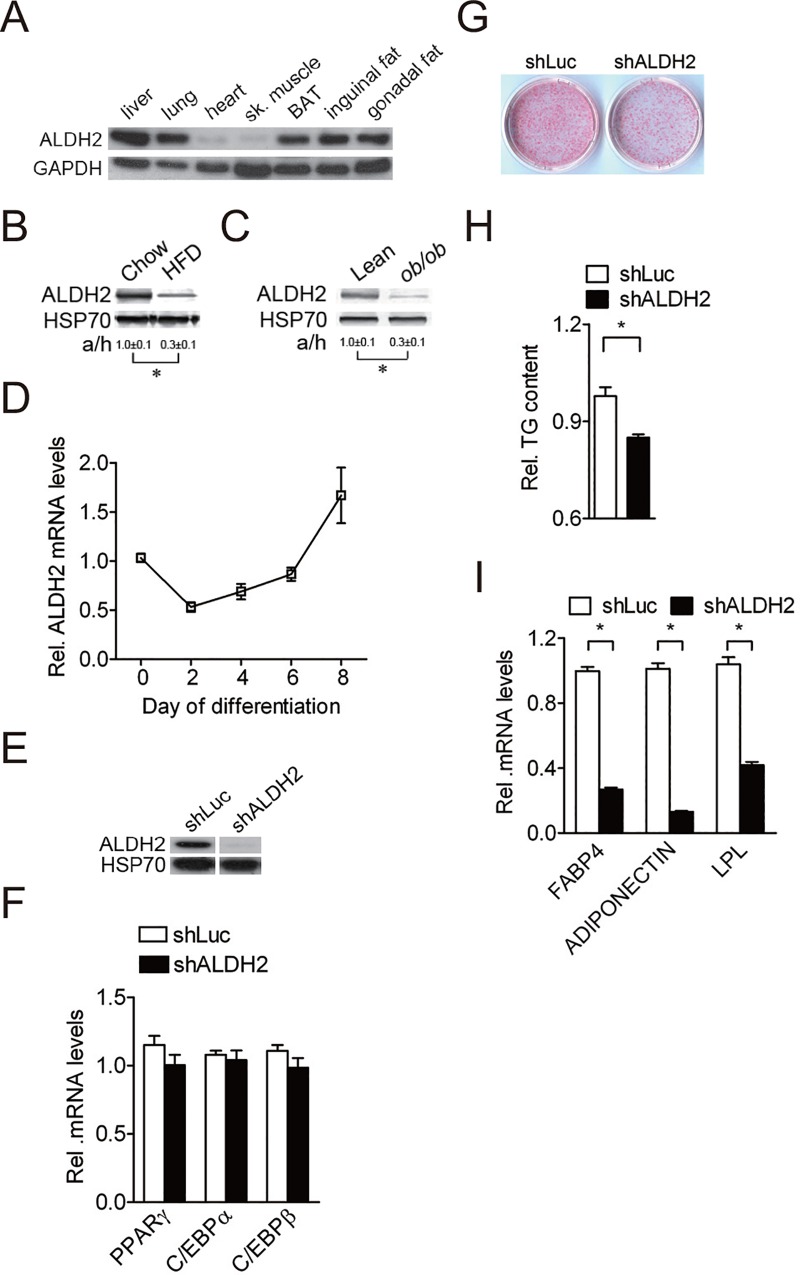
ALDH2 is a positive regulator of adipocyte differentiation. *A*, Tissue distribution of ALDH2 protein from 12-wk-old male mice C57BL/6 mice (n = 3 independent experiments). The results shown are representative of an individual experiment. *B*, Expression of ALDH2 protein from adipose tissue of 12-wk-old male chow/HFD-fed mice (n = 4 independent experiments). 4-wk-old mice C57BL/6 mice were randomly divided into two groups: a control group, which was fed a chow diet (Chow), and a high-fat diet group, which was fed a high-fat diet (HFD). The results shown are representative of an individual experiment. The ratio of intensity of bands corresponding to target protein per loading control was analyzed by densitometer software. The numbers indicate the means ± SE. * P < 0.05 versus chow diet group. *C*, Expression of ALDH2 protein from white adipose tissue of 12-wk-old male lean and *ob/ob* mice (n = 4 independent experiments). The results shown are representative of an individual experiment. The ratio of intensity of bands corresponding to target protein per loading control was analyzed by densitometer software. The numbers indicate the means ± SE. * P < 0.05 versus lean mice. *D*, Expression of ALDH2 mRNA during induced 3T3-L1 adipocyte differentiation. Data are shown as mean ± SE from 3 independent experiments. *E*, Efficiency of shRNA control (shLuc) and ALDH2 shRNA (shALDH2) targeted on mouse ALDH2 protein levels of 3T3-L1 cells. Data are shown as mean ± SE from 3 independent experiments. *F*, Expression of adipogenic transcription factor genes (PPARγ, C/EBPα and C/EBPβ) in shRNA control (shLuc) and ALDH2-knockdown cells after 2 day induction. Data are shown as mean ± SE from 3 independent experiments. *G*, Oil-Red O staining of shRNA control and ALDH2-knockdown cells during adipocyte differentiation (n = 3 independent experiments). The results shown are representative of an individual experiment. *H*, Quantification of Oil-Red O in shLuc control and ALDH2-knockdown cells during adipocyte differentiation. Data are shown as mean ± SE from 3 independent experiments. * P < 0.05 versus shLuc. *I*, Expression of adipogenic genes (FABP4, ADIPONECTIN and LPL) in shRNA control and ALDH2-knockdown cells during adipocyte differentiation. Data are shown as mean ± SE from 3 independent experiments. * P < 0.05 versus shLuc.

To explore the physiological function of ALDH2, we first examined the tissue expression pattern of ALDH2. ALDH2 protein is broadly expressed in several tissues, including lung, liver, intestine, brown adipose tissue and white adipose tissue (Fig 1A). We also investigated whether ALDH2 levels were altered in murine models of obesity. We found that ALDH2 levels were decreased in white adipose tissue of high-fat diet (HFD)-fed mice (Fig 1B) and of *ob/ob* mice (Fig 1C). Furthermore, ALDH2 mRNA levels greatly increased during induced adipogenesis of 3T3-L1 cells (Fig 1D). These results indicate that ALDH2 may be involved in adipocyte differentiation.

To determine whether ALDH2 is required for adipocyte differentiation, 3T3-L1 preadipocytes were infected with lentivirus carrying ALDH2 shRNA and were then induced for adipocyte differentiation. shRNAs targeting ALDH2 efficiently attenuated ALDH2 protein levels in 3T3-L1 preadipocytes throughout adipogenesis (Fig 1E and S1A Fig). Silencing of ALDH2 in 3T3-L1 preadipocytes did not affect cell proliferation (S1B Fig). The expression of transcription factors involved in adipogenesis, including PPARγ, C/EBPα and C/EBPβ were not affected after 2 day induction by ALDH2 shRNA (Fig 1F). After 8-day adipogenic induction, adipocyte differentiation was reduced in ALDH2 knockdown cells as compared with shRNA control cells (shLuc) (Fig 1G), accompanied by reduced intracellular triglyceride levels (Fig 1H). Consistently, the levels of markers for adipocyte differentiation, including adipocyte fatty acid binding protein (FABP4), ADIPONECTIN and lipoprotein lipase (LPL) all decreased in ALDH2 knockdown cells as compared with shRNA control cells after hormonal induction (Fig 1I). By contrast, the expression of preadipocyte marker, PREF-1 was greater in ALDH2 knockdown cells than shRNA control cells, indicating adipocyte differentiation was impaired in ALDH2 knockdown cells (S1C Fig). Taken together, these results demonstrate ALDH2 positively modulates adipocyte differentiation.

### Activation of ALDH2 promotes adipocyte differentiation

**Fig 2:**
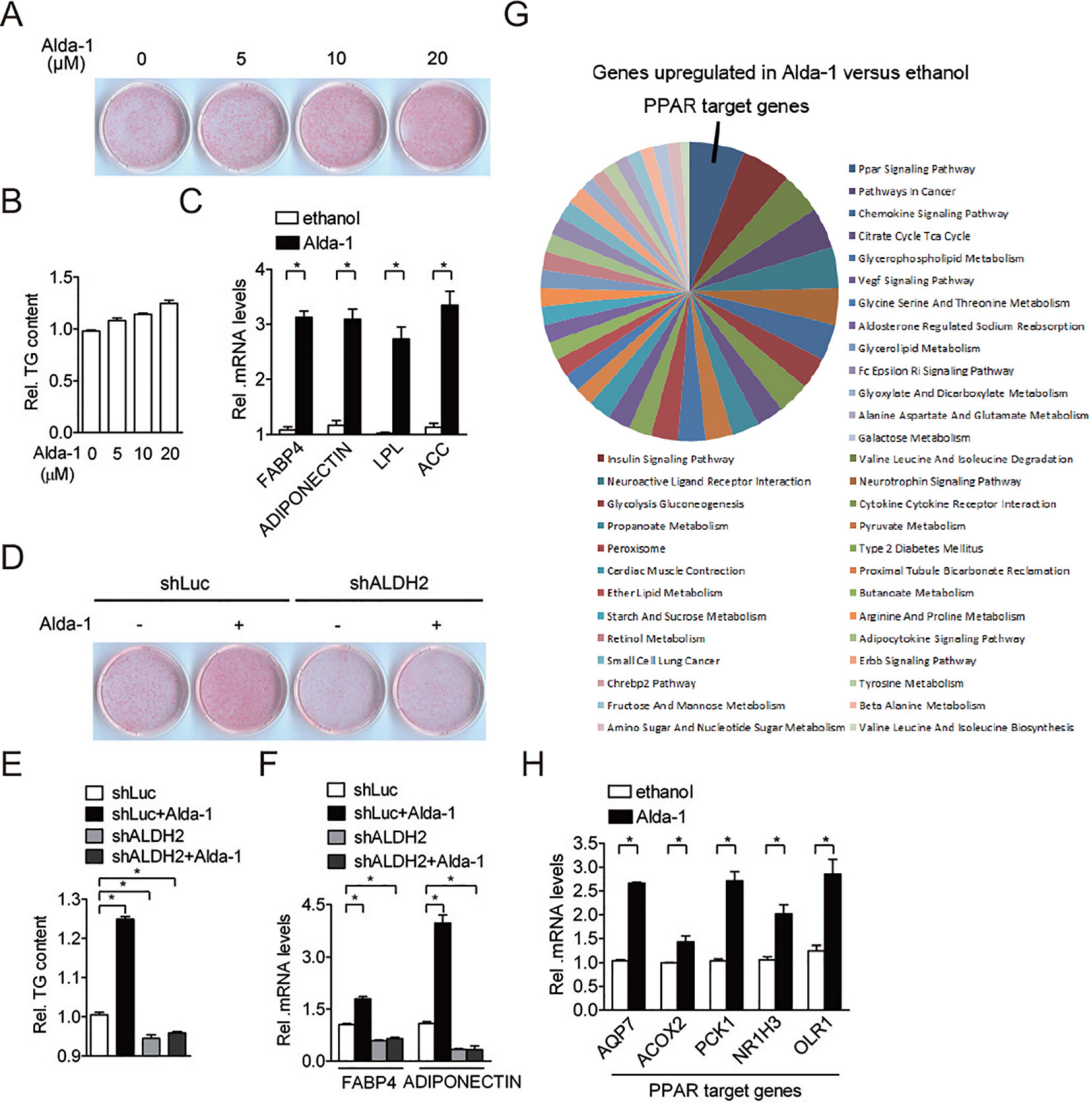
Activation of ALDH2 promotes adipocyte differentiation. *A*, Effect of various dose of Alda-1 on adipogenesis. The results shown are representative of an individual experiment. 3T3-L1 preadipocytes were maintained in induction medium with or without Alda-1 for 2 days. After 8 day of adipogenic stimulation, cells on the plates were stained with Oil-Red O. *B*, Quantification of Oil-Red O dye in ethanol-treated and Alda-1-treated cells after 8 day induction. Data are shown as mean ± SE from 3 independent experiments. *C*, Expression of adipogenic gene (FABP4, ADIPONECTIN, LPL and ACC) expression in ethanol-treated and Alda-1-treated cells after 8 day induction. Data are shown as mean ± SE from 3 independent experiments. * P < 0.05 versus ethanol. *D*, shRNA control (shLuc) and ALDH2-knockdown preadipocytes were maintained in induction medium with or without 10 μM Alda-1 treatment for 2 days. After 8 day of adipogenic stimulation, cells on the plates were stained with Oil-Red O. *E*, Quantification of Oil-Red O dye in shRNA control (shLuc) and ALDH2-knockdown preadipocytes with or without 10 μM Alda-1 treatment after 8 day induction. *F*, Determination of adipogenic gene (FABP4 and ADIPONECTIN) expression in shRNA control (shLuc) and ALDH2-knockdown preadipocytes with or without 10 μM Alda-1 treatment after 8 day induction. Data are shown as mean ± SE from 4 independent experiments. * P < 0.05 versus shLuc. *G*, Functional classification of representative microarray data for the most significant upregulated genes in Alda-1-treated versus ethanol-treated 3T3-L1 adipocytes. Percentage of genes sharing common biological processes is presented. Data are shown from 3 independent experiments. *H*, Verification of microarray data by quantitative reverse transcription-PCR. PPAR target genes (AQP7, ACOX2, PCK1, NR1H3 and OLR1) identified from microarray data were selected for verification. Data are shown as mean ± SE from 3 independent experiments. * P < 0.05 versus ethanol.

To investigate whether ALDH2 activity plays a role in regulating adipogenesis, 3T3-L1 preadipocytes were treated with the ALDH2 activator, Alda-1, and were then induced for adipocyte differentiation. The expression of adipogenic transcription factors was not affected by Alda-1 treatment after 2 day induction (S1D Fig). The Alda-1 solvent, ethanol did not alter the degree of adipogenic differentiation in 3T3-L1 preadipocytes and C3H10T1/2 cells (S1E and S1F Fig). However, adipocyte differentiation was enhanced in a dose-dependent manner after 8-day adipogenic induction (Fig 2A), which was accompanied by an increase in intracellular triglyceride content (Fig 2B) and adipocyte differentiation marker expression (Fig 2C). The adipogenic effect of Alda-1 was also observed in C3H10T1/2 cells (S2A–S2C Fig). By contrast, Alda-1 did not further promote adipogenesis in the differentiated adipocytes (S2D Fig), implying that Alda-1 mainly regulates adipogenesis during the early phase of differentiation. To determine whether ALDH2 is required for Alda-1-mediated pro-adipogenic effect, we treated shRNA control cells and ALDH2 knockdown cells with or without Alda-1. Similar to the previous results, Alda-1 significantly promoted adipocyte differentiation in cells treated with control shRNA (Fig 2D). Conversely, knockdown of ALDH2 suppressed adipocyte differentiation in 3T3-L1 cells (Fig 2D). However, the pro-adipogenic effect of Alda-1 was remarkably abolished when ALDH2 protein was silenced in 3T3-L1 preadipocytes (Fig 2D). Similar results were also found for intracellular triglyceride content (Fig 2E) and adipogenic marker gene expression (Fig 2F). To further explore the role of Alda-1 in adipocytes, we performed a whole-genome microarray analyses. The results of 3 independent microarrays showed that Alda-1 treatment resulted in upregulation of several transcripts encoding proteins involved in metabolism; the majority of these transcripts code for proteins involved in PPAR signaling pathway (Fig 2G). Five genes from a total 12 upregulated PPAR target genes identified by microarray analysis were confirmed by the quantitative reverse transcription-PCR (Fig 2H). Taken together, these results demonstrate that ALDH2 activity is associated with PPAR signaling pathway.

### Adipogenesis is regulated by PKCε-ALDH2 axis

**Fig 3:**
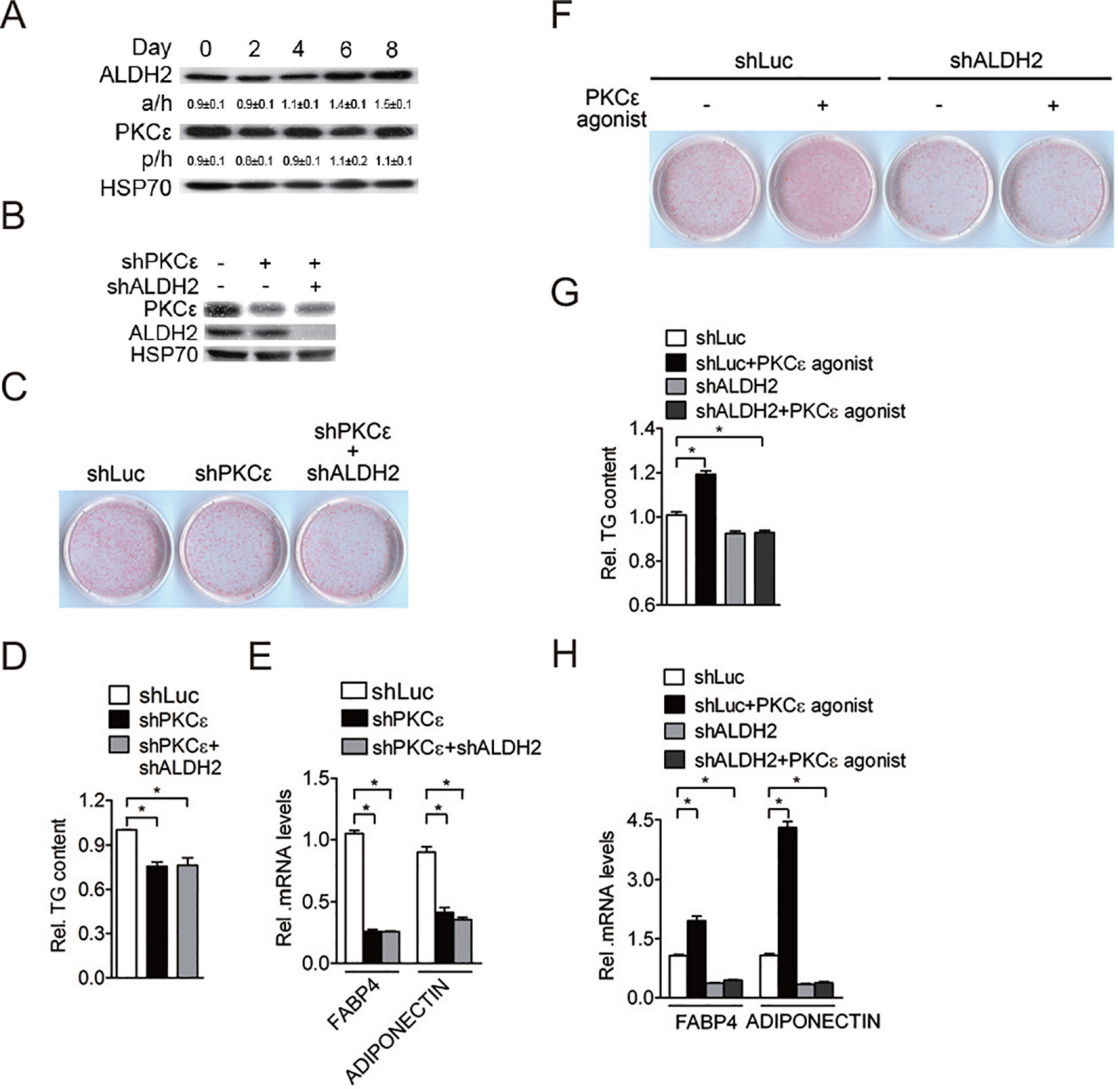
Adipogenesis is regulated by PKCε-ALDH2 axis. *A*, Expression of ALDH2 and PKCε protein during induced 3T3-L1 adipocyte differentiation (n = 3 independent experiments). The results shown are representative of an individual experiment. The ratio of intensity of bands corresponding to target protein per loading control was analyzed by densitometer software. The numbers indicate the means ± SE. *B*, Efficiency of shRNA control (shLuc), PKCε shRNA and ALDH2 shRNA targeted on mouse PKCε and ALDH2 protein levels of 3T3-L1 cells (n = 3 independent experiments). The results shown are representative of an individual experiment. *C*, 3T3-L1 preadipocytes expressing indicated lentiviral vectors were maintained in induction medium for 2 days. After 8 day of adipogenic stimulation, cells on the plates were stained with Oil-Red O. *D*, Quantification of Oil-Red O dye in shRNA control, PKCε-knockdown and double knockdown of PKCε and ALDH2 preadipocytes after 8 day induction. *E*, Determination of adipogenic gene (FABP4 and ADIPONECTIN) expression in shRNA control, PKCε-knockdown and double knockdown of PKCε and ALDH2 preadipocytes after 8 day induction. Data are shown as mean ± SE from 3 independent experiments. * P < 0.05 versus shLuc. *F*, shRNA control and ALDH2-knockdown preadipocytes were maintained in induction medium with or without 1 μM PKCε agonist for 2 days. After 8 day of adipogenic stimulation, cells on the plates were stained with Oil-Red O. *G*, Quantification of Oil-Red O dye in shRNA control, PKCε-knockdown and double knockdown of PKCε and ALDH2 preadipocytes with or without 1 μM PKCε agonist treatment after 8 day induction. *H*, Determination of adipogenic gene (FABP4 and ADIPONECTIN) expression in shRNA control, PKCε-knockdown and double knockdown of PKCε and ALDH2 preadipocytes with or without 1 μM PKCε agonist treatment after 8 day induction. Data are shown as mean ± SE from 3 independent experiments. * P < 0.05 versus shLuc.

Protein kinase C epsilon (PKCε) is an upstream activator of ALDH2 activity by direct ALDH2 phosphorylation [9]. To determine whether PKCε is also involved in adipogenesis, the expression of PKCε during induced adipogenesis of 3T3-L1 cells was determined. We found that PKCε levels remained unchanged during adipocyte differentiation, while ALDH2 levels were gradually increased (Fig 3A). We further tested whether PKCε affects adipocyte differentiation. 3T3-L1 preadipocytes were infected with lentivirus carrying PKCε shRNA and were then induced for adipocyte differentiation. shRNAs targeting PKCε efficiently attenuated PKCε protein levels in 3T3-L1 preadipocytes and ALDH2 shRNA knocked down 3T3-L1 preadipocytes (Fig 3B), whereas adipogenic transcription factors were not affected by PKCε shRNA (S3A Fig). After adipogenic induction, adipogenesis was reduced in PKCε knockdown cells compared with shRNA control cells (shLuc) (Fig 3C), accompanied by reduced intracellular triglyceride levels (Fig 3D) and adipogenic marker gene expression (Fig 3E). Importantly, adipocyte differentiation was not further reduced when 3T3-L1 preadipocytes were simultaneously infected with PKCε shRNA and ALDH2 shRNA (Fig 3C–3E), suggesting that PKCε and ALDH2 positively regulate adipogenesis through a common pathway.

To examine whether PKCε activation can induce adipogenesis, 3T3-L1 preadipocytes were treated with the PKCε agonist and were then induced for adipocyte differentiation. After exposure to a hormonal cocktail, adipocyte differentiation was remarkably enhanced (Fig 3F), accompanied by increased intracellular triglyceride levels (Fig 3G). The PKCε, ALDH2 and adipogenic transcription factor expression was not affected by PKCε agonist treatment (S3B and S3C Fig), while terminal adipocyte differentiation marker expression was increased (Fig 3H). However, the pro-adipogenic effect of PKCε agonist was completely abolished when ALDH2 expression was silenced in 3T3-L1 preadipocytes (Fig 3F–3H). Taken together, these results suggest that PKCε-mediated activation of adipogenesis is dependent on ALDH2 activity.

### PKCε-ALDH2 interaction regulates PPARγ activity

**Fig 4:**
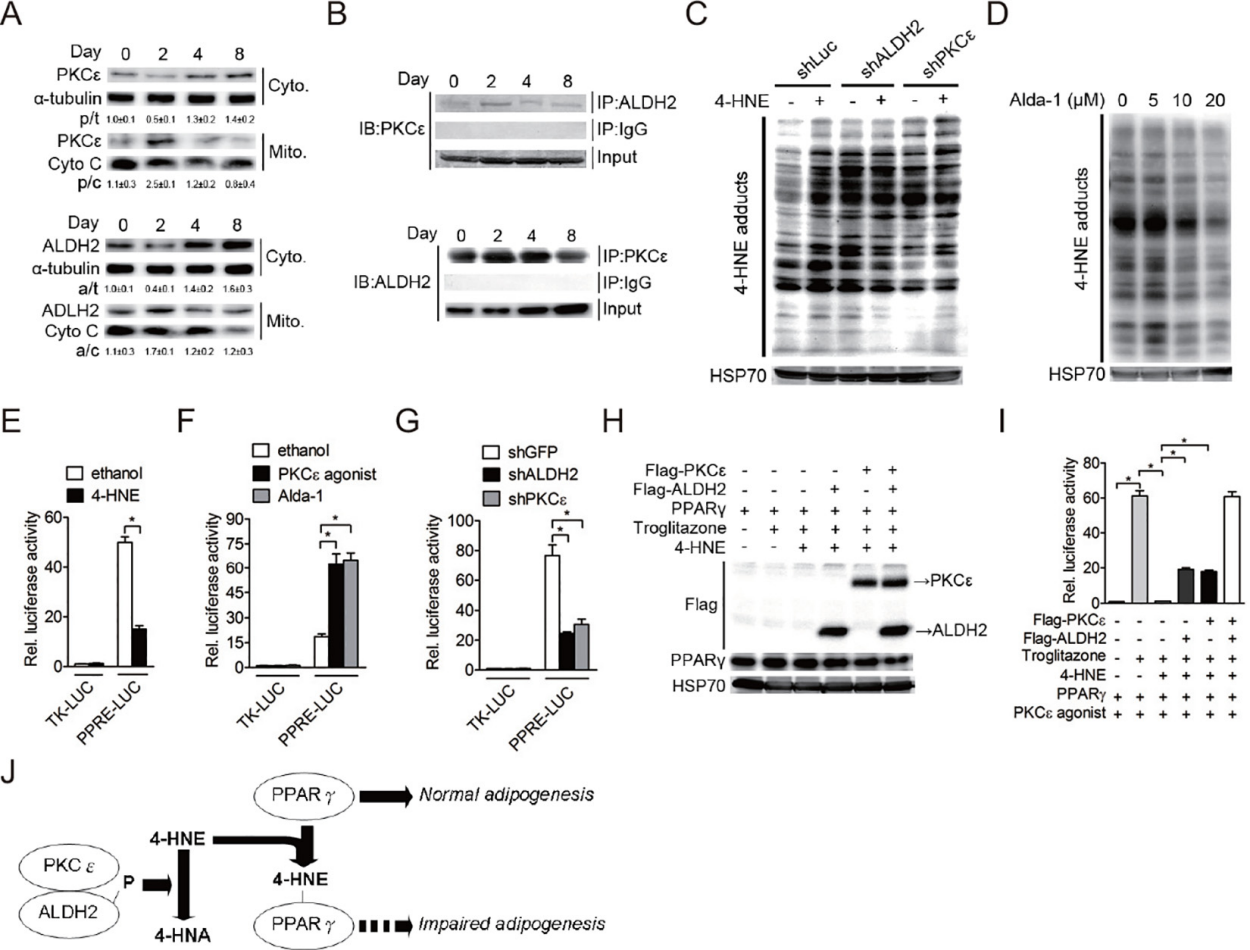
PKCε-ALDH2 interaction involves in regulation of PPARγ activity. *A*, Cytoplasmic and mitochondrial distribution of PKCε and ALDH2 protein throughout the differentiation process (n = 3 independent experiments). The results shown are representative of an individual experiment. The ratio of intensity of bands corresponding to target protein per loading control was analyzed by densitometer software. The numbers indicate the means ± SE. *B*, PKCε coimmunoprecipitates with ALDH2 during adipocyte differentiation. Cell extracts harvested from differentiating adipocytes were immunoprecipitated (IP) with either IgG, ALDH2 or PKCε antibody. The immunoblots (IB) were probed for either PKCε or ALDH2 antibody. Input is shown in the lower panels (n = 4 independent experiments). The results shown are representative of an individual experiment. *C*, shRNA control, ALDH2-knockdown and PKCε-knockdown preadipocytes were treated with or without 10 μM 4-hydroxynonenal (4-HNE) for 24 hours. Total cell extracts were harvested and analyzed by immunoblotting with anti-4-HNE antibody (n = 4 independent experiments). The results shown are representative of an individual experiment. *D*, Effect of Alda-1 on 4-HNE formation. 3T3-L1 preadipocytes were maintained in induction medium with various dose of Alda-1 for 2 days. After 8 day of adipogenic stimulation, total cell extracts were harvested and analyzed by immunoblotting with anti-4-HNE antibody (n = 4 independent experiments). The results shown are representative of an individual experiment. *E*, 4-HNE attenuates PPRE-driven luciferase activity in differentiating 3T3-L1 cells. Differentiating 3T3-L1 cells were transfected with reporter vectors (TK-LUC and PPRE-LUC) for 24 hour and then incubated cells in induction medium with or without treatments (10 μM 4-HNE) for 24 hour. The activity of firefly luciferase was determined and normalized to the activity of renilla luciferase. Data are shown as mean ± SE from 4 independent experiments. * P < 0.05 versus ethanol. *F*, PKCε agonist and Alda-1 trigger PPRE-driven luciferase activity in differentiating 3T3-L1 cells. Differentiating 3T3-L1 cells were transfected with reporter vectors (TK-LUC and PPRE-LUC) for 24 hour and then incubated cells in induction medium with or without treatments (1 μM PKCε agonist and 10 μM Alda-1) for 2 days. The activity of firefly luciferase was determined and normalized to the activity of renilla luciferase. Data are shown as mean ± SE from 4 independent experiments. * P < 0.05 versus ethanol. *G*, Silencing of ALDH2 or PKCε reduces PPRE-driven luciferase activity in differentiating 3T3-L1 cells. Differentiating shGFP, shALDH2 and shPKCε cells were transfected with reporter vectors (TK-LUC and PPRE-LUC) for 24 hour. The activity of firefly luciferase was determined and normalized to the activity of renilla luciferase. Data are shown as mean ± SE from 4 independent experiments. * P < 0.05 versus shGFP. *H*, PKCε-ALDH2 pathway potentiates PPARγ transcriptional activity. 293 cells were transiently transfected with expression vectors (Flag-ALDH2, Flag-PKCε and CMX-GAL4-PPARγ) and UAS_G_×4-TK-LUC reporter plasmid for 24 hour and then incubated cells in growth medium with 1 μM PKCε agonist, 1 μM troglitazone and 10 μM 4-HNE for 2 days (n = 3 independent experiments). The results shown are representative of an individual experiment. *I*, The activity of firefly luciferase was determined and normalized to the activity of renilla luciferase. Data are shown as mean ± SE from 4 independent experiments. * P < 0.05 versus control cells. *J*, Proposed model for the mechanism by which PKCε-ALDH2 interaction involves in regulation of PPARγ activity by 4-HNE metabolism.

Several studies demonstrated that PKCε translocates from cytoplasm into the mitochondria and then physically interact with mitochondrial ALDH2, thereby increasing ALDH2 activity by phosphorylation [9, 15]. However, the subcellular localization and consequences of PKCε translocation during adipocyte differentiation had not been determined. Thus, we analyzed PKCε and ALDH2 protein levels in the cytoplasm and mitochondria during induced adipogenesis of 3T3-L1 cells. The results showed that cytoplasmic PKCε protein was significantly decreased after hormonal induction, while mitochondrial PKCε protein was increased coincidentally at day 2 and then declines during terminal differentiation (Fig 4A). Interestingly, similar results were also found for ALDH2 protein (Fig 4A), indicating ALDH2 and PKCε protein translocate simultaneously from cytoplasm to mitochondria in early phase of adipogenesis. We also found that PKCε-ALDH2 interaction is remarkably increased to a peak at day 2 after initiation of differentiation and then declines (Fig 4B). These findings indicate that subcellular localization in mitochondria and interaction of PKCε with ALDH2 plays a role during adipocyte differentiation in a time-dependent manner.

ALDH2 is a mitochondrial enzyme that catalyzes the metabolism of 4-hydroxynonenal (4-HNE). We therefore determined whether aberrant adipogenesis in ALDH2 or PKCε knockdown cells was due to increased intracellular 4-HNE levels. As shown in Fig 4C, silencing of ALDH2 or PKCε expression in 3T3-L1 preadipocytes caused an increase in intracellular 4-HNE-protein adducts formation to levels similar to those induced following 4-HNE treatment (Fig 4C). Furthermore, Alda-1 or PKCε agonist significantly alleviated 4-HNE-protein adducts formation in 3T3-L1 adipocytes (Fig 4D and S4 Fig), indicating that PKCε-ALDH2 pathway is required for 4-HNE metabolism in adipocytes. 4-HNE adduction to protein affects their structure and functions [4]. PPARγ is a master transcription factor involved in adipogenesis and its transcriptional activity is highly regulated by several post-translational modifications [16] and our whole-genome microarray showed that many PPAR target genes were induced after Alda-1 treatment (Fig 2G). Therefore, we investigate whether ALDH2 activation regulates PPARγ transcriptional activity. We transfected a PPARγ responsive element (PPRE)-driven luciferase plasmid into differentiating 3T3-L1 preadipocytes and then treated these cells with 4-HNE, Alda-1 and PKCε agonist. We found that 4-HNE treatment significantly impaired the PPRE-driven luciferase activity on day 2 of differentiation (Fig 4E), while ALDH2 and PKCε agonist significantly activated the PPRE-driven luciferase activity in the early stage of adipogenesis (Fig 4F). Consistently, PPRE-driven luciferase activity was reduced in ALDH2- or PKCε-knockdown cells (Fig 4G).

To further confirm our hypothesis that PKCε-ALDH2 interaction directly regulates PPARγ activity through metabolism of 4-HNE, we co-transfected 293 cells with Flag-PKCε, Flag-ALDH2, CMX-GAL4-PPARγ, and UAS_G_×4-TK-LUC plasmids (Fig 4H) and then treated these cells with PPARγ agonist, 4-HNE and PKCε agonist. As expected, PPARγ agonist, troglitazone significantly activated PPARγ-driven luciferase activity, but this effect was remarkably reduced by 4-HNE treatment (Fig 4I). Furthermore, forced expression of PKCε or ALDH2 protein partially abolished 4-HNE-mediated inhibition of PPARγ transcriptional activity (Fig 4I). Importantly, co-expression of PKCε and ALDH2 protein remarkably enhanced PPARγ-driven luciferase in the presence of 4-HNE treatment (Fig 4I). Finally, we also preliminarily found that PPARγ proteins are highly modified by 4-HNE before hormonal induction and then declines during adipogenesis (S5 Fig). Taken together, these results demonstrate that PKCε-ALDH2 interaction is involved in adipogenesis through regulation of PPARγ activity and PKCε-ALDH2 axis may indirectly affect PPARγ activity by metabolism of 4-HNE (Fig 4J).

## Discussion

The major finding of this study is that we identified ALDH2 as a positive regulator of adipocyte differentiation; increased ALDH2 expression or enzyme activity in 3T3-L1 cells significantly accelerated adipocyte differentiation. We also found that ALDH2 upstream regulator, PKCε, is involved in adipogenesis. Similar to ALDH2, increased PKCε expression or activity is highly correlated with increased adipocyte differentiation. Furthermore, the PKCε-ALDH2 interaction is required for a proper adipocyte differentiation *via* regulation of PPARγ transcriptional activity.

The ALDH2 is an important enzyme that metabolizes acetaldehyde to acetic acid, and also detoxifies other reactive aldehydes, such as 4-HNE. ALDH2 is phosphorylated by PKCε and its activity is negatively correlated with infarct size in animal model of cardiac ischemia [9]. Activation of PKCε in the ischemic heart with PKCε agonist increased ALDH2 activity and leads to reduction in infarct size [9]. Consistently, administration of ALDH2 agonist, Alda-1, ameliorated 4-HNE concentration and infarct size in animals [9]. Furthermore, activation of ALDH2 prevents ischemic stroke by reducing 4-HNE levels [17]. These findings demonstrate that ALDH2 is a key mediator of endogenous cytoprotection against ischemia injury.

The role of ALDH2 or aldehydes in the pathogenesis of diabetes and obesity has not been fully understood. Here, we found that ALDH2 expression in white adipose tissue was reduced in high-fat diet-induced and genetic *ob/ob* obese mice models. This is consistent with observations that many genes encoding mitochondrial enzymes, including ALDH2 are down-regulated in genetically obese mice [11]. Previous study demonstrated that PPARγ induces ALDH2 expression by direct binding to specific sites in the ALDH2 promoter [18]. Consistent with that observation, we also found that, similar to PPARγ, expression of ALDH2 in 3T3-L1 preadipocytes, was increased during the course of induced adipocyte differentiation, indicating that ALDH2 is associated with fat cell differentiation. Furthermore, silencing of ALDH2 or PKCε in 3T3-L1 preadipocytes negatively affects adipocyte differentiation, demonstrating that ALDH2 and PKCε activities are required for adipogenesis.

Adipocytes are particularly sensitive to oxidative stress due to a high percentage of lipids in their cytosol. 4-HNE, a bioreactive aldehyde, is a major product of lipid peroxidation [19]. Plasma 4-HNE concentration is significantly increased in obese patients compared with healthy control [3]. Intracellular lipid content in adipocytes is positively associated with 4-HNE levels [6]. 4-HNE rapidly forms adduct with proteins by reacting with the side chains of cysteine and histidine residues which leads to alternation of protein functions [4]. Adipose proteins from high-fat diet-fed mice are highly carbonylated with 4-HNE [4] and treatment of differentiating preadipocytes with 4-HNE promotes 4-HNE-protein adducts formation, alters adipokine expression, attenuates subsequent adipocyte differentiation and increases free fatty acid release [20, 6]. These results indicate that intracellular 4-HNE accumulation in adipocytes contributes to dysregulation of adipocyte differentiation and functions. Moreover, our current study further consolidates the key role of ALDH2 during adipogenesis. We found that knockdown of ALDH2 or PKCε in 3T3-L1 preadipocytes accelerated 4-HNE-protein adducts formation, thereby reducing adipocyte differentiation. These findings are consistent with other studies [20, 6] that intracellular 4-HNE accumulation in 3T3-L1 preadipocytes leads to an impaired adipocyte differentiation. In addition, we found that pro-adipogenic effect of Alda-1 or PKCε agonist was abolished when ALDH2 expression was silenced. These results support previous reports [8, 9], showing that ALDH2 is a key mediator in signaling transduction by clearing intracellular 4-HNE levels. We also observe that translocation and interaction of PKCε and ALDH2 is increased to a peak in early phase of adipogenesis and then declines, while 4-HNE levels were gradually increased during adipocyte differentiation, implicating PKCε-ALDH2 pathway mainly involves in early stage of adipogenesis. Indeed, activation of PKCε-ALDH2 pathway either by overexpressed ALDH2 or PKCε agonist induces PPARγ transcriptional activity and enhances subsequent differentiation. The pro-adipogenic effect of Alda-1 was not observed in the differentiated adipocytes, indicating that activation of ALDH2 mainly regulates PPARγ transcriptional activity during the early phase of differentiation. Consistently, it has been demonstrated that PKCε is involved in promoting the early phase of adipogenesis in 3T3-F442A preadipocytes [21]. Furthermore, elevated and direct PKCε-ALDH2 interaction promotes PPARγ-dependent transcription. Together, these findings demonstrate that PKCε-ALDH2 pathway involves adipogenesis through regulation of PPARγ transcriptional activity.

Several mechanisms have been proposed by which 4-HNE can cause an aberrant adipocyte functions. Exposure of adipocytes to exogenous 4-HNE results in decreased adiponectin secretion by increasing adiponectin protein degradation [22]. 4-HNE exposure also promotes lipolysis in adipocyte through activation of cAMP/PKA signal pathway [6]. Finally, 4-HNE treatment alters adipogenic, lipolytic gene expression and protein oxidation in differentiating adipocytes [20]. Here, we found that PPARγ protein levels were increased after hormonal induction and were dynamically modified by 4-HNE during adipogenesis. Interestingly, the ALDH2 protein expression and the level of 4-HNE-modifed PPARγ proteins were negatively correlated during adipocyte differentiation. We also demonstrate that 4-HNE is able to impair PPARγ transcriptional activity, thereby attenuating PPARγ-target gene expression. In addition, activation of PKCε-ALDH2 pathway ameliorates 4-HNE-induced dysregulation of PPARγ. These results imply that elevated ALDH2 protein levels or activity during adipogenesis may reduce 4-HNE-modifed PPARγ protein levels, thereby facilitating a normal adipocyte differentiation. However, many questions remain to be further answered, for example, whether transcription activity of PPARγ is altered in 4-HNE-mediated carbonylation and which amino acids are modified by 4-HNE and functional.

In conclusion, we provide evidence that PKCε-ALDH2 pathway positively regulates adipocyte differentiation by regulation of PPARγ transcriptional activity. ALDH2 expression in white adipose tissue is negatively correlated with obesity. Obesity is due to abnormal fat accumulation in adipose tissue. Here, we demonstrate that ALDH2 is required for a normal adipogenesis. Thus, modulation of ALDH2 expression and/or its activity in white adipose tissue might provide a novel avenue in treating obesity and related metabolic disorders.

## Supporting Information

S1 Fig Effect of ALDH2 shRNA on ALDH2 protein levels, cell proliferation and expression of PREF-1 in 3T3-L1 cells during adipogenesis and ethanol does not affect adipocyte differentiation. *A*, Efficiency of shRNA control (shLuc) and ALDH2 shRNA (shALDH2) targeted on mouse ALDH2 protein levels of 3T3-L1 cells during adipogenesis (n = 3 independent experiments). The results shown are representative of an individual experiment. *B*, Relative proliferation rates of shLuc control and ALDH2-knockdown 3T3-L1 preadipocytes. Cell proliferation was evaluated by MTS assay after 24 h and 48 h seeding. Data are shown as mean ± SE from 3 independent experiments. *C*, Expression of PREF-1 in shRNA control and ALDH2-knockdown cells at day 8 of differentiation. Data are shown as mean ± SE from 3 independent experiments. * P < 0.05 versus shLuc. *D*, Expression of adipogenic transcription factor genes (PPARγ, C/EBPα and C/EBPβ) in ethanol-treated and Alda-1-treated cells after 2 day induction. Data are shown as mean ± SE from 3 independent experiments. *E*, Effect of ethanol on adipogenic differentiation of 3T3-L1 preadipocytes and C3H10T1/2 cells. 3T3-L1 preadipocytes and C3H10T1/2 cells were maintained in induction medium with or without ethanol for 2 days. After 8 day of adipogenic stimulation, cells on the plates were stained with Oil-Red O (n = 3 independent experiments). *F*, Quantification of Oil-Red O dye in ethanol-treated 3T3-L1 preadipocytes and C3H10T1/2 cells after 8 day induction. Data are shown as mean ± SE from 3 independent experiments.
(TIF)

S2 Fig Activation of ALDH2 promotes adipogenic differentiation of C3H10T1/2 cells. *A*, Effect of Alda-1 on adipogenic differentiation of C3H10T1/2 cells. C3H10T1/2 cells were maintained in induction medium with or without Alda-1 for 2 days. After 8 day of adipogenic stimulation, cells on the plates were stained with Oil-Red O (n = 3 independent experiments). *B*, Quantification of Oil-Red O dye in Alda-1-treated C3H10T1/2 cells after 8 day induction. Data are shown as mean ± SE from 3 independent experiments. * P < 0.05 versus blank. *C*, Expression of adipogenic gene (FABP4 and ADIPONECTIN) expression in Alda-1-treated cells after 8 day induction. Data are shown as mean ± SE from 3 independent experiments. * P < 0.05 versus blank. *D*, Expression of adipogenic gene (FABP4 and ADIPONECTIN) in Alda-1-treated fully-differentiated 3T3-L1 adipocytes. Data are shown as mean ± SE from 3 independent experiments.
(TIF)

S3 Fig Knockdown of PKCε expression or treatment of PKCε agonist in 3T3-L1 preadipocytes does not affect adipogenic transcription factor expression. *A*, Expression of adipogenic transcription factor genes (PPARγ, C/EBPα and C/EBPβ) in shRNA control (shLuc) and PKCε-knockdown cells after 2 day induction. *B*, Effect of various dose of PKCε agonist on ALDH2 and PKCε protein levels (n = 3 independent experiments). The results shown are representative of an individual experiment. *C*, Expression of adipogenic transcription factor genes (PPARγ, C/EBPα and C/EBPβ) in control and PKCε agnoist-treated cells after 2 day induction. Data are shown as mean ± SE from 4 independent experiments.
(TIF)

S4 Fig Effect of PKCε agonist on 4-HNE formation. 3T3-L1 preadipocytes were maintained in induction medium with various dose of PKCε agonist for 2 days. After 8 day of adipogenic stimulation, total cell extracts were harvested and analyzed by immunoblotting with anti-4-HNE antibody (n = 3 independent experiments). The results shown are representative of an individual experiment.
(TIF)

S5 Fig PPARγ proteins are dynamically modified by 4-HNE during adipogenesis. Cell extracts harvested from differentiating adipocytes were immunoprecipitated (IP) with PPARγ antibody. The immunoblots (IB) were probed for either 4-HNE or PPARγ antibody (n = 3 independent experiments). The results shown are representative of an individual experiment.
(TIF)
