# Supplementary figures and images for: PKC-ALDH2 Pathway Plays a Novel Role in Adipocyte Differentiation
Source: PLoS One. 2016 Aug 30;11(8):e0161993. doi: 10.1371/journal.pone.0161993 (PMC5004862; doi:10.1371/journal.pone.0161993)

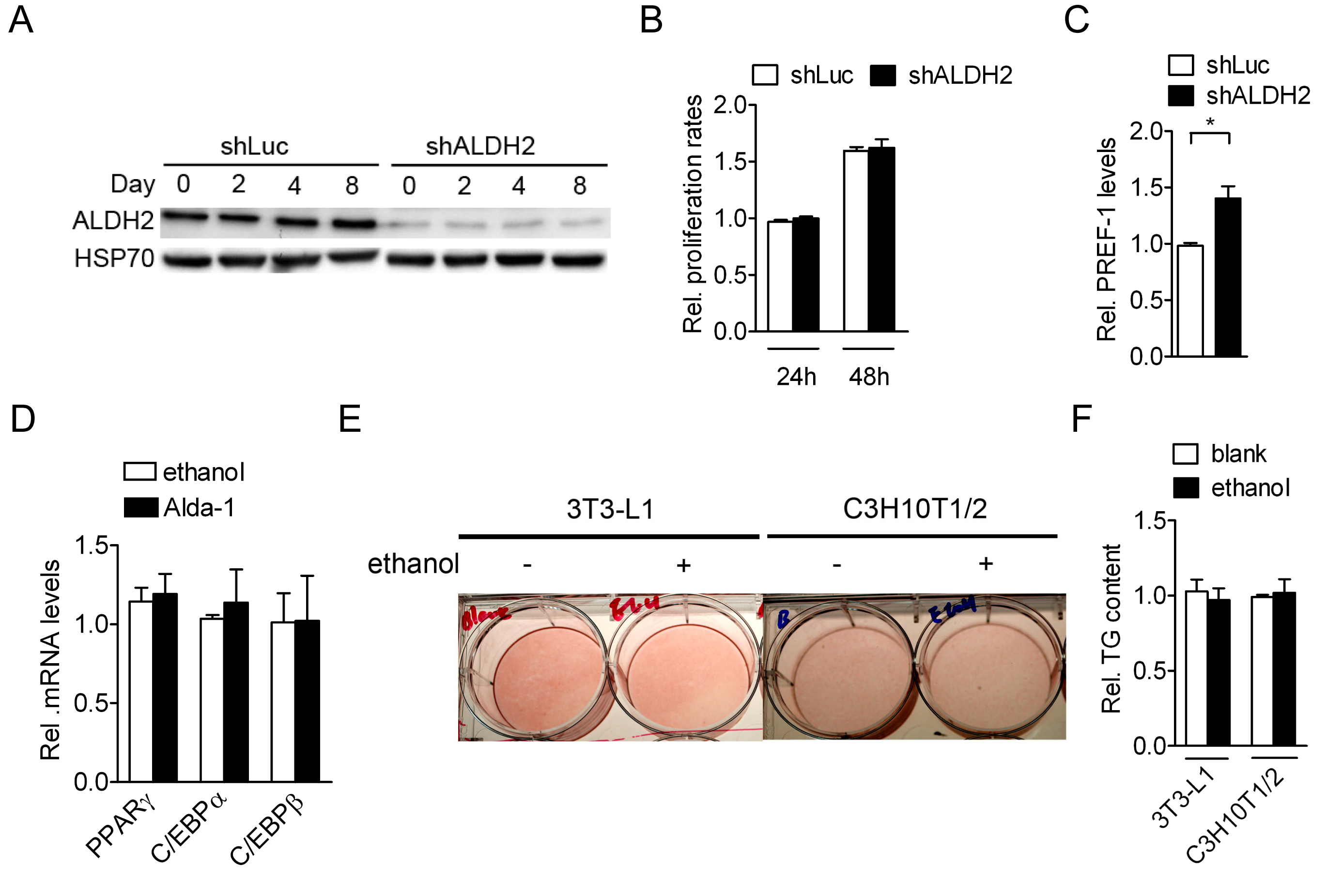

Supplement: S1 Fig — A, Efficiency of shRNA control (shLuc) and ALDH2 shRNA (shALDH2) targeted on mouse ALDH2 protein levels of 3T3-L1 cells during adipogenesis (n = 3 independent experiments). The results shown are representative of an individual experiment. B, Relative proliferation rates of shLuc control and ALDH2-knockdown 3T3-L1 preadipocytes. Cell proliferation was evaluated by MTS assay after 24 h and 48 h seeding. Data are shown as mean ± SE from 3 independent experiments. C, Expression of PREF-1 in shRNA control and ALDH2-knockdown cells at day 8 of differentiation. Data are shown as mean ± SE from 3 independent experiments. * P < 0.05 versus shLuc. D, Expression of adipogenic transcription factor genes (PPARγ, C/EBPα and C/EBPβ) in ethanol-treated and Alda-1-treated cells after 2 day induction. Data are shown as mean ± SE from 3 independent experiments. E, Effect of ethanol on adipogenic differentiation of 3T3-L1 preadipocytes and C3H10T1/2 cells. 3T3-L1 preadipocytes and C3H10T1/2 cells were maintained in induction medium with or without ethanol for 2 days. After 8 day of adipogenic stimulation, cells on the plates were stained with Oil-Red O (n = 3 independent experiments). F, Quantification of Oil-Red O dye in ethanol-treated 3T3-L1 preadipocytes and C3H10T1/2 cells after 8 day induction. Data are shown as mean ± SE from 3 independent experiments. (TIF) [file pone.0161993.s001.tif]

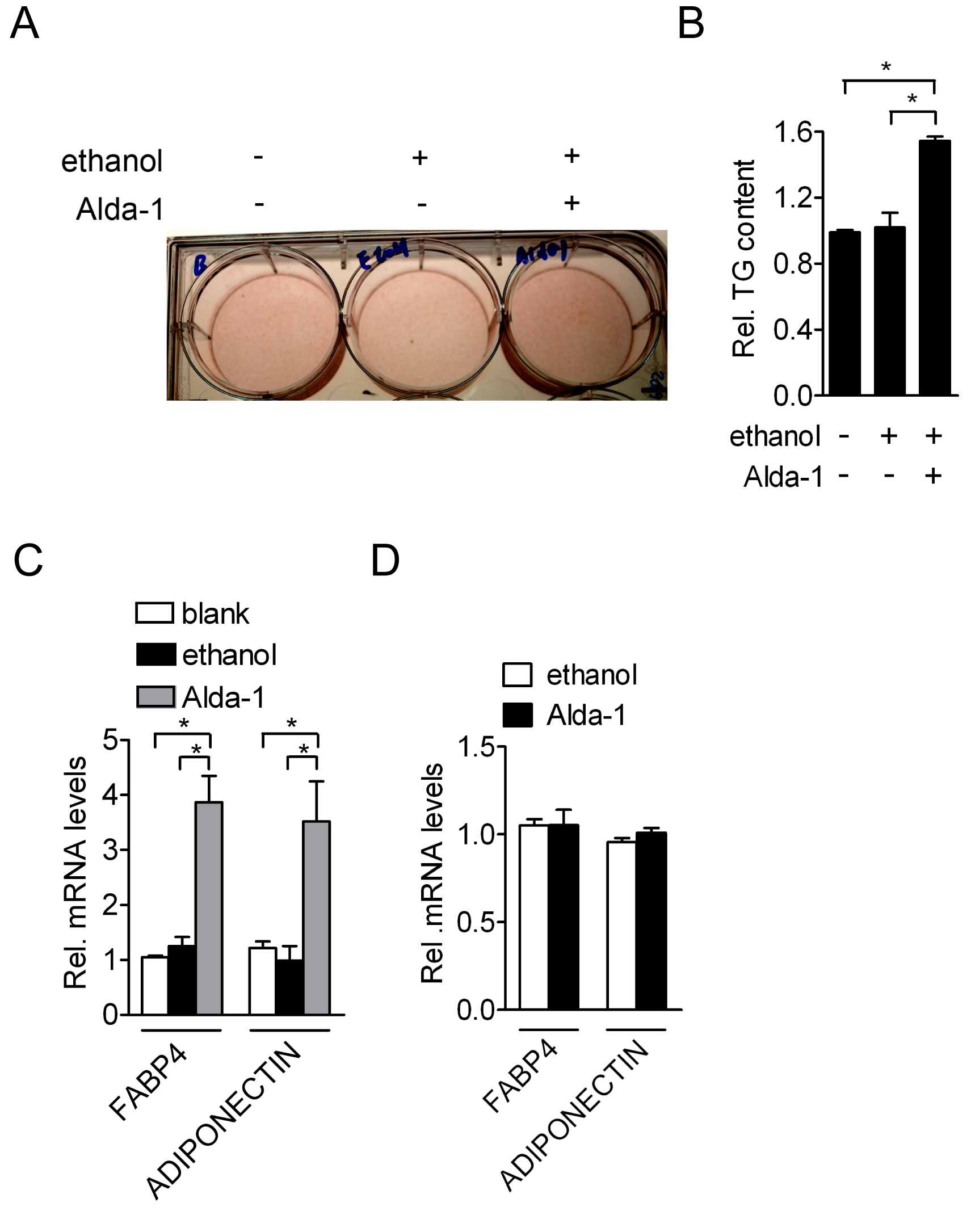

Supplement: S2 Fig — A, Effect of Alda-1 on adipogenic differentiation of C3H10T1/2 cells. C3H10T1/2 cells were maintained in induction medium with or without Alda-1 for 2 days. After 8 day of adipogenic stimulation, cells on the plates were stained with Oil-Red O (n = 3 independent experiments). B, Quantification of Oil-Red O dye in Alda-1-treated C3H10T1/2 cells after 8 day induction. Data are shown as mean ± SE from 3 independent experiments. * P < 0.05 versus blank. C, Expression of adipogenic gene (FABP4 and ADIPONECTIN) expression in Alda-1-treated cells after 8 day induction. Data are shown as mean ± SE from 3 independent experiments. * P < 0.05 versus blank. D, Expression of adipogenic gene (FABP4 and ADIPONECTIN) in Alda-1-treated fully-differentiated 3T3-L1 adipocytes. Data are shown as mean ± SE from 3 independent experiments. (TIF) [file pone.0161993.s002.tif]

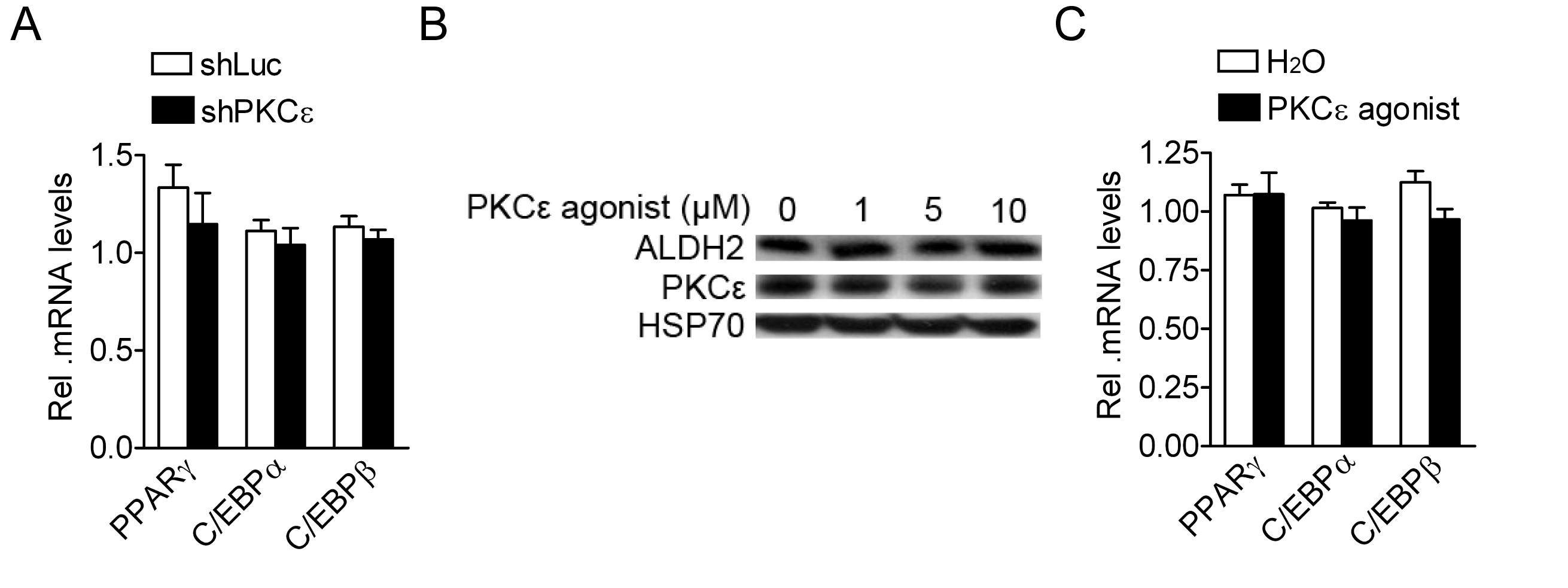

Supplement: S3 Fig — A, Expression of adipogenic transcription factor genes (PPARγ, C/EBPα and C/EBPβ) in shRNA control (shLuc) and PKCε-knockdown cells after 2 day induction. B, Effect of various dose of PKCε agonist on ALDH2 and PKCε protein levels (n = 3 independent experiments). The results shown are representative of an individual experiment. C, Expression of adipogenic transcription factor genes (PPARγ, C/EBPα and C/EBPβ) in control and PKCε agnoist-treated cells after 2 day induction. Data are shown as mean ± SE from 4 independent experiments. (TIF) [file pone.0161993.s003.tif]

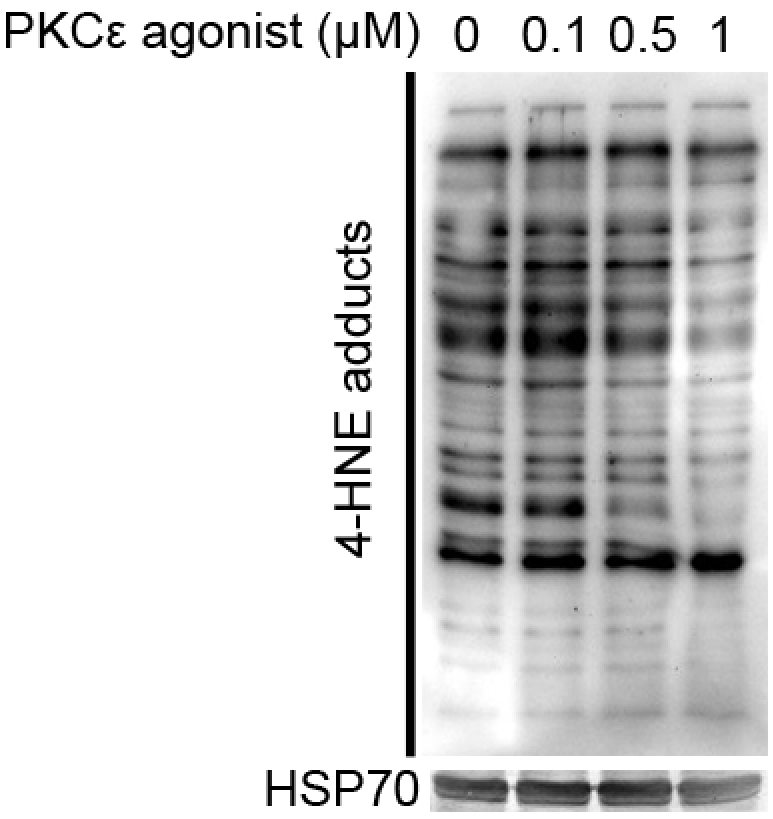

Supplement: S4 Fig — 3T3-L1 preadipocytes were maintained in induction medium with various dose of PKCε agonist for 2 days. After 8 day of adipogenic stimulation, total cell extracts were harvested and analyzed by immunoblotting with anti-4-HNE antibody (n = 3 independent experiments). The results shown are representative of an individual experiment. (TIF) [file pone.0161993.s004.tif]

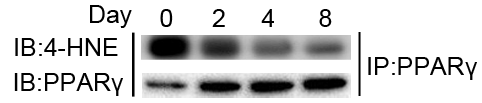

Supplement: S5 Fig — Cell extracts harvested from differentiating adipocytes were immunoprecipitated (IP) with PPARγ antibody. The immunoblots (IB) were probed for either 4-HNE or PPARγ antibody (n = 3 independent experiments). The results shown are representative of an individual experiment. (TIF) [file pone.0161993.s005.tif]
